# Hodge Decomposition of Single-Cell RNA Velocity

**DOI:** 10.1021/acs.jcim.4c00132

**Published:** 2024-04-04

**Authors:** Zhe Su, Yiying Tong, Guo-Wei Wei

**Affiliations:** † Department of Mathematics, Michigan State University, East Lansing, Michigan 48824, United States; ‡ Department of Computer Science and Engineering, Michigan State University, East Lansing, Michigan 48824, United States; § Department of Electrical and Computer Engineering, Michigan State University, East Lansing, Michigan 48824, United States; ∥ Department of Biochemistry and Molecular Biology, Michigan State University, East Lansing, Michigan 48824, United States

## Abstract

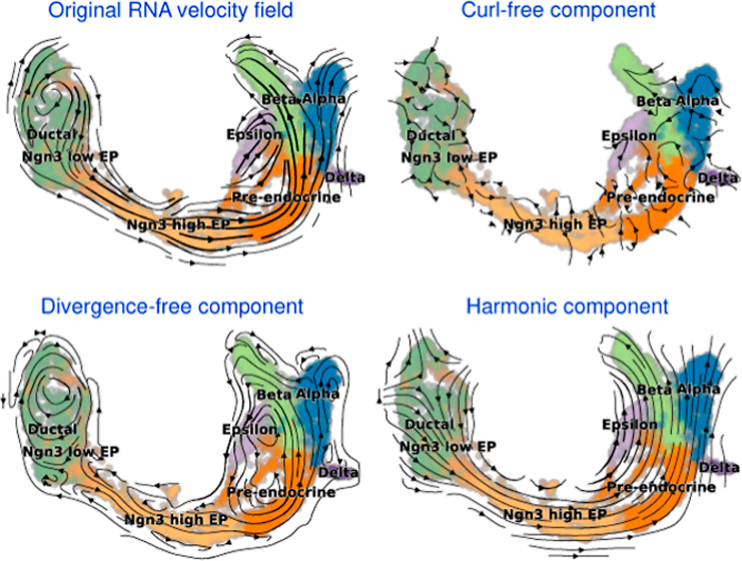

RNA velocity has the ability to capture the cell dynamic information in the biological processes; yet, a comprehensive analysis of the cell state transitions and their associated chemical and biological processes remains a gap. In this work, we provide the Hodge decomposition, coupled with discrete exterior calculus (DEC), to unveil cell dynamics by examining the decomposed curl-free, divergence-free, and harmonic components of the RNA velocity field in a low dimensional representation, such as a UMAP or a t-SNE representation. Decomposition results show that the decomposed components distinctly reveal key cell dynamic features such as cell cycle, bifurcation, and cell lineage differentiation, regardless of the choice of the low-dimensional representations. The consistency across different representations demonstrates that the Hodge decomposition is a reliable and robust way to extract these cell dynamic features, offering unique analysis and insightful visualization of single-cell RNA velocity fields.

## 1 Introduction

Single-cell RNA sequencing (scRNA-seq), with its growing availability of public data resources, has witnessed rapid advances over the past decade. This technology enables the study of gene expression at the resolution of individual cells. Due to the distinct gene signatures of cells, researchers are able to distinguish different cell types, identify unknown/rare cells, study the cellular responses to external stimuli (such as disease or drugs), and investigate the cell dynamics in gene expressions like cell cycle or cell differentiation. Various tools have been developed to delve into the gene signatures of cells, aiding in the understanding of cell types, states, and behaviors in biological research.^1−4^ Mathematical approaches based on algebraic topology^5,6^ and differential geometry^7,8^ have also been developed for analyzing scRNA-seq data.

The scRNaseq approach, however, provides only static snapshots of cellular states at the moment of measurement and poses a challenge in revealing dynamic transitions between different cell states. To address the issue, La Manno et al. in^9^ proposed a RNA velocity model, called the steady-state model, using ordinary differential equations (ODEs) with some assumptions on the transcription, splicing, and degradation rates of genes. The model utilizes the unspliced and spliced mRNA abundances to analyze the dynamic information on cells from their gene expressions, but does not require any prior information about the relationships between different cell states. The high-dimensional RNA velocity, defined as the time derivative of the spliced abundance, was inferred and then visualized as vector fields in a low-dimensional representation of cells, usually obtained using uniform manifold approximation and projection (UMAP)^10^ and t-distributed stochastic neighbor embedding (t-SNE).^11^ The visualizations of the velocity flows on cell points could reflect directly the cell lineage information between different cell states by examining the flow directions. It can also be used to identify the cell cycle, which is usually represented by vortices formed by the velocity flow. The success of the RNA velocity model has captured the attention of many researchers and led to various interesting works that generalize the RNA velocity model, such as scVelo,^12^ dynamo,^13^ CellRank,^14^ etc. Despite significant progress, there is still a notable gap in our understanding of cell state transitions and the underlying biological processes. This serves as motivation for us to create mathematical frameworks dedicated to the study of RNA velocity fields. In Section 2.1, we give a basic introduction of two primary RNA velocity models—the steady-state model and the dynamic model and also illustrate how the velocity flows in the low-dimensional representation of cell points can be obtained.

The analysis of vector fields has a long history in many different fields, such as fluid dynamics,^15,16^ astrophysics and geophysics,^17−19^ graphics and visualization,^20,21^ computer vision and robotics,^22,23^ and imaging.^24^ A widely used powerful tool to study the vector fields in low-dimensional Euclidean spaces is the Helmholtz–Hodge decomposition (HHD),^25^ which decomposes a vector field into a curl-free, a divergence-free, and a harmonic part, and thus can be used for extracting important features. Depending on the applications, one could study each component separately to identify important properties like irrotationality (curl-free) or incompressibility (divergence-free) of the vector field. These properties are particularly important in applications like modeling and analyzing fluids, such as water and smoke,^26^ which requires that the flow be divergence-free, and biomolecules.^27^ In HHD, one of the most critical aspects is the consideration of boundary conditions, which ensures the orthogonality and the uniqueness of the decomposition. The importance of the orthogonality of these components has been discussed in.^28^ A variety of boundary conditions have been explored, and many numerical algorithms have been developed to implement the HHD in the discrete case.^28,29^ The importance of this decomposition motivated us to study these extracted components of the velocity flow on cell points obtained by using the RNA velocity models for scRNA-seq data.

To apply HHD, one way is to proceed through Hodge decomposition. The Hodge decomposition is defined for differential *k*-forms on a Riemannian manifold, and it decomposes a differential *k*-forms into three *L*^2^-orthogonal components, including an exact form, a coexact form, and a harmonic field. It was first proposed by Hodge^30^ for finite-dimensional compact manifolds without any boundary, and then fully extended to compact manifolds with boundary in,^31^ incorporating the Dirichlet and the Neumann boundary conditions. Details regarding these boundary conditions are listed in Section 4. The framework of differential forms serves as a generalization of vector calculus of lower-dimensional Euclidean spaces to Riemannian manifolds. This leads to a correspondence between the HHD for vector fields and the Hodge decomposition for differential forms (eq 4.12) when considering bounded domains in the 2-dim or 3-dim Euclidean space with respect to the standard Euclidean metric. The three components—the exact part, the coexact part, and the harmonic part of a differential form, correspond directly to the curl-free part, the divergence-free part, and the harmonic part of the corresponding vector field, respectively. The computations of these orthogonal differential forms in the Hodge decomposition are various.^32,33^ One of the widely used powerful tools is the discrete exterior calculus (DEC),^32^ which is a discrete counterpart of the smooth exterior calculus. It is simple and efficient by using essentially just the matrix algebra. Depending on different applications, the manifold can be represented as a tetrahedral mesh in a Euclidean space^21^ or as a bounded domain in a regular Cartesian grid.^34^ By utilization of the differential form representation of a vector field, the Hodge decomposition can be applied through DEC to extract the curl-free, divergent-free, and harmonic components of the vector field.

Distinct from the Hodge decomposition, the graph Hodge decomposition,^35^ based on cell complex connectivity, has been applied to single-cell data.^36^ In this context, trajectories of single-cell data are visualized as sparse diffusion graphs in which paths could be used to identify specific cell states or cell differentiation. However, the results only reflect the connections between cell points but do not provide any physically relevant components, such as curl-free, divergence-free, and harmonic components, as described in the Hodge decomposition defined on differential forms. For a comparison between the related combinatorial Laplacian and Hodge Laplacian, refer to.^34^

Recently, the natural Helmholtz–Hodge decomposition^37^ has been utilized on scRNA-seq data to gain insights into cell differentiation and cell cycle on the decomposed curl-free and divergence-free components of the RNA velocity fields.^38^ The decomposition process involves solving the Poisson equation to obtain potentials, followed by applying the gradient and rotation operators to calculate the curl-free and divergence-free components. However, the decomposed components lack orthogonality within the bounded domain, and the algorithm is computationally expensive.

In this work, we employ the Hodge decomposition, utilizing DEC, to study the velocity flow on cell points, calculated using the RNA velocity model in a low-dimensional representation for certain scRNA-seq data sets. By utilizing the FRI density function, as described in Section 4.4, we transform the discrete cell points to a continuous manifold covering all cell points in a regular Cartesian grid with boundary given by an isocurve of the FRI density function. We create a vector field on this bounded domain by using the weighted average of the velocities on cell points and then perform the Hodge decomposition to extract its curl-free, divergence-free, and harmonic components. These components are then analyzed to identify and unveil features corresponding to known biological processes of cells. The results show that the important features, such as cell cycle, bifurcation, and cell lineage information, could be effectively extracted from the original velocity flow and studied on its components. Furthermore, the results demonstrate consistency across different representations, making the Hodge decomposition a reliable way for providing varied visualizations of the original velocity field. Like applications in other fields, these components offer opportunities for further exploration in understanding the cell dynamics in the biological process.

## 2 Results

### 2.1 Overview of Single-Cell RNA Velocity

Transcription is a process of making an RNA copy of a gene’s DNA sequence that carries the gene’s protein information. For most eukaryotic genes, the resulting RNA, referred to as precursor mRNA (unspliced mRNA), contains not just the coding regions but also the noncoding regions. Therefore, it must be processed before it becomes a mature mRNA (spliced mRNA) to be ready for translation into proteins. This process is known as RNA splicing. In this process, the noncoding regions of RNA, called introns, will be removed, and the coding regions of RNA, called exons, will be kept. The final mRNA consists only of the exons and then goes through the degradation process in the final step. The abundances of the unspliced mRNA and the spliced mRNA can be quantified using reads mapping the exonic and intronic regions of genes. Denote by *u* the abundance of the unspliced mRNA and by *s* the abundance of the spliced mRNA. Let α(*t*), β(*t*), and γ(*t*) be the rates of transcription, splicing, and degradation, respectively. The RNA velocity model that describes the transcriptional dynamics for a gene is given by a system of ODEs^9^
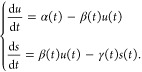
2.1

These two rate equations describe how the abundances of unspliced mRNA molecules *u*, and spliced molecules *s* evolve for each single gene, independently. The RNA velocity is defined to be the rate of change of the abundance of the spliced mRNA, i.e., . To apply the RNA velocity model, three parameters α(*t*), β(*t*), and γ(*t*) need to be inferred. Currently, there are two primary approaches to estimating RNA velocity—the steady-state model^9^ and the dynamic model.^12^ The steady-state model assumes that the splicing dynamics have reached their steady states, i.e., α(*t*) = α, γ(*t*) = γ, and β(*t*) = 1, with a common splicing rate across genes and estimates the parameters with a linear regression fit, while the dynamical model generalizes the steady-state model and assumes constant gene-specific splicing rate β and degradation rate γ with two transcription rates α each for induction and repression and uses an expectation-maximization framework to estimate parameters.

The RNA velocity analysis of single cells enables a prediction of the temporal dynamics of cells from gene expression. One could use the combination of velocities across genes to estimate the future state of an individual cell. However, the space of cells with thousands of genes can be seen as a low-dimensional manifold embedded in a high-dimensional space, and thus, dimensionality reduction techniques need to be used to project the cell points and velocity vectors to a low-dimensional space, where the low-dimensional representation of the data will be analyzed and visualized. Commonly used dimensionality reduction techniques include principal component analysis, UMAP, and t-SNE. To project the velocities onto a lower-dimensional space, the transition probabilities of cell-to-cell transitions need to be estimated. For each velocity vector, the most likely cell transition aligned with its direction can be identified using the cosine correlation between the cell-to-cell transitions and the velocity vector.^9,12^ In the low-dimensional representation, the velocities on cell points can be easily visualized and studied, showing the dynamics of cells in the biological process. Various RNA velocity methods^12−14^ have generalized these two models due to the success of the RNA velocity model.

In this paper, we select UMAP as the primary representation method, as UMAP, compared with other commonly used dimensional reduction methods, has shown fast run time, high reproducibility, and meaningful organization of cell clusters for large single-cell data sets.^39^ However, to demonstrate the robustness of our method, we present similar results using t-SNE in Section 3.

### 2.2 Hodge Decomposition on scRNA-seq Velocity Fields

**Figure 1:**
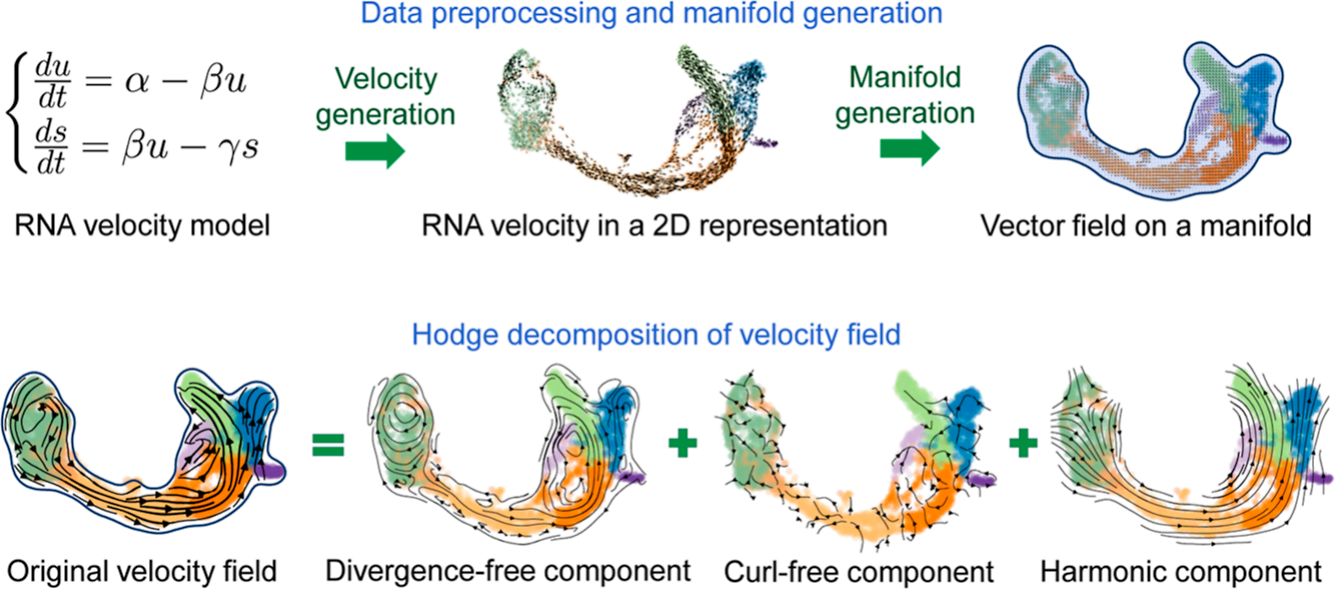
Illustration of the Hodge decomposition of single-cell RNA velocity field in a 2D representation. Here, *u* and *s* denote the abundances of the unspliced mRNA and the spliced mRNA, respectively. α, β, and γ are the transcription rate, the splicing rate, and the degradation rate, respectively. The original velocity field was created as the streamline plot of the velocity on a manifold.

To present the Hodge decomposition results, we considered three different scRNA-seq data sets: the endocrine pancreas sample data set included in the scVelo package,^12^ the cell cycle data set^40^ and the developing mouse hippocampus scRNA-seq data set from the original RNA velocity paper.^9^ Following Section 4.4, each data set was preprocessed and projected to a 2D UMAP representation. The velocity field on a bounded manifold in a 2D Cartesian grid was then constructed by considering the weighted average of the RNA velocities on cell points for the vector field calculation and by using the FRI density function for manifold generation. In this paper, we employed the dynamic model^12^ for the calculation of RNA velocities. Finally, Hodge decomposition was applied to the vector fields on these bounded manifolds, generating their curl-free, divergence-free, and harmonic components. A schematic plot of procedure is given in Figure 1.

#### 2.2.1 Endocrine Pancreas scRNA-seq Velocity Field

**Figure 2:**
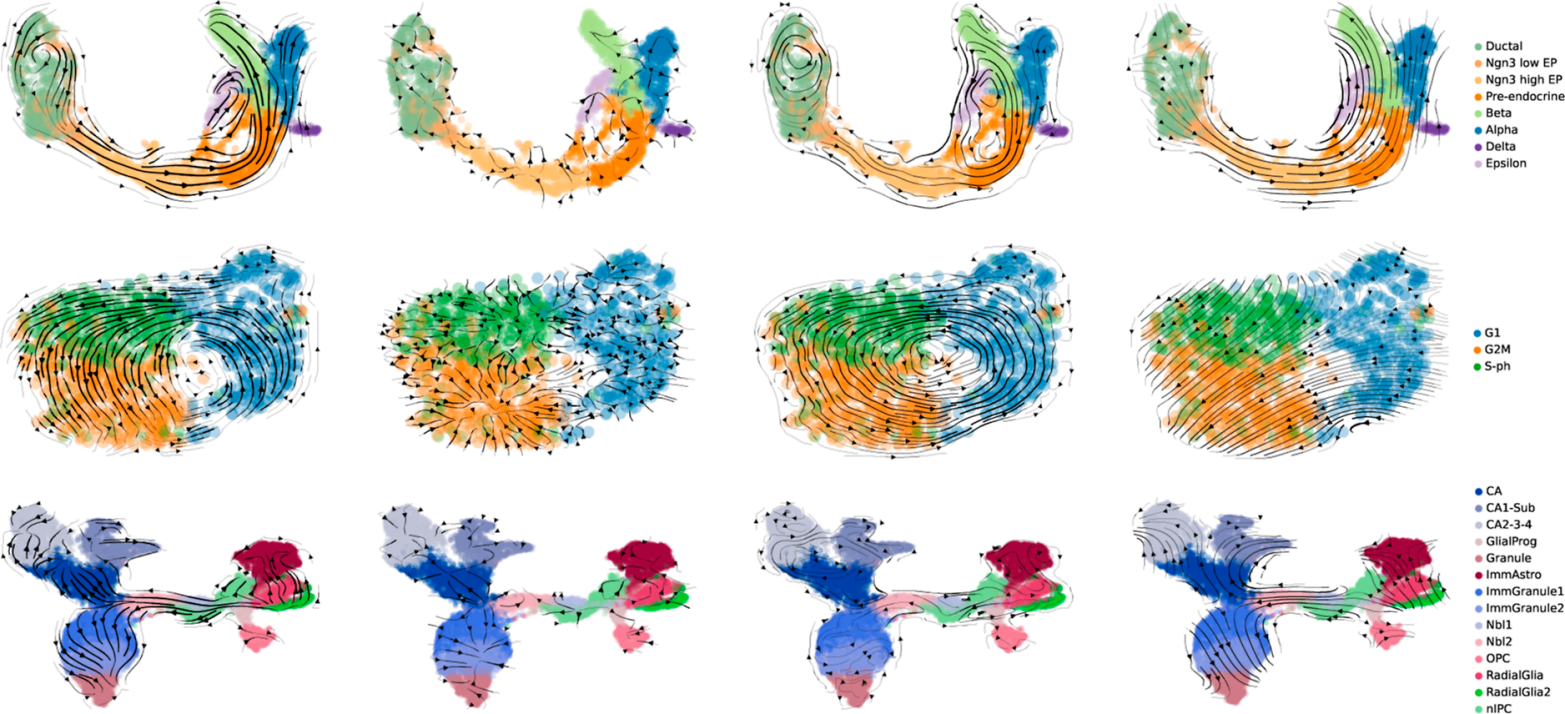
Hodge decomposition of velocity fields in 2D UMAP representations for the pancreatic endocrinogenesis data set (top row), the cell cycle phase data set (middle row), and the developing mouse hippocampus data set (bottom row). Each row, from left to right, presents the original velocity field, its curl-free component, its divergence-free component, and its harmonic component. Here, color represents different cell types or cell phases.

The endocrine pancreas sample data set^12^ describes the transient lineages in endocrine development in the mouse pancreas, containing 3696 cells and 27,998 genes. These cells are categorized into eight different cell types with lineages to four terminal states: alpha, beta, delta, and epsilon cells. After preprocessing the data based on parameters specified in the scVelo tutorial notebook, the final data set for analysis includes 3696 cells and the top 2000 high variance genes. The RNA velocity dynamic model was then applied to the data, with velocities calculated on cell points in a 2D UMAP representation. In the first row of Figure 2, we present on the left the velocity flow on cell points as streamlines in the 2D UMAP representation. One can easily see that the streamlines delineate the cycling population of Ductal cells and the endocrine differentiation along the velocity flow. Visualizations of the curl-free, divergence-free, and harmonic components of the corresponding velocity field on a bounded manifold *M* covering all cell points are illustrated in the first row of Figure 2 as well.

The circular shape in Ductal cells in the divergence-free component, notably, captures the cell cycle process in the population of endocrine progenitors. Additionally, the presence of the saddle point in the Ductal cells in the harmonic component indicates that these cells are in an unstable state undergoing a bifurcation process, which is potentially attributed to the cells being involved in either the cell cycle or the cell differentiation process. Note that circular shapes can also be seen across the other cell types in the divergence-free component. However, the streamlines in the harmonic component related to these cell types are unidirectional, suggesting that they do not correspond to cell cycles. Otherwise, the cells would undergo a bifurcation process, and there will be saddle points shown in the harmonic component within these cell types. The cell lineage information is clearly presented in the harmonic component, given by the overall direction of the streamlines from the progenitors to the four final terminal states.

It should be noted that circular shapes appearing near the boundary in the divergence-free component are most likely artifacts. This occurrence arises as we enforce the tangential boundary condition on the divergence-free part, i.e., all boundary vectors of the divergence-free part must be tangent to the boundary of the domain, which might result in a backflow of the vector field and finally form these circular shapes with small velocity magnitudes, when the harmonic component is separated.

#### 2.2.2 Cell Cycle scRNA-seq Velocity Field

The cell cycle is a series of events that happen in a cell, including phase G1 (cell growth), S (DNA replication), G2 (growth and preparation for cell division), and M (cell division). In addition, there is a stage called the G1 checkpoint, also known as the restriction checkpoint, which is a regulatory point for a cell to choose whether or not to divide, or enter the cell cycle. Cell cycle is a directional process that the cells undergo from one phase to another in the order mentioned above. For the second example, we consider the cell cycle data set, adapted from^40^ containing 1067 cell points. This data set was filtered by selecting the top 5000 variance genes with minimum shared counts of each gene being set to 10 and then normalized and log-transformed. In the second row of Figure 2, we show the streamline plot of the original velocity field on cell points in a 2D UMAP representation and its decomposed components obtained by using the Hodge decomposition. The streamlines in the original velocity field clearly illustrate the cell phase direction and also show a bifurcation process that happened in the G1 phase, characterizing the separation of G1 and G1 checkpoint as indicated in.^41^

Due to the separation of G1 and G1-checkpoint in G1 phase and the cell cycle process from G1 to S. The separation of G1 and G1-checkpoint in G1 phase and the cell cycle process from G1 to S is evident in the harmonic component by examining the saddle point located in G1 phase, which shows an unstable state of cells going through the bifurcation process. One can also see the overall direction trend of the streamline plot in the harmonic component from G1 to G2M. The divergence-free component clearly captures the cell cycle process and its direction, visualized in a circular shape over all cell phases.

#### 2.2.3 Developing Mouse Hippocampus scRNA-seq Velocity Field

The developing mouse hippocampus scRNA-seq data set, used in the original RNA velocity paper,^9^ consists of 18,140 cells and 2160 genes. The developing process of cells is multibranching, with the origin of the lineage tree given by radial glia and five terminal stages given by astrocytes (IMMAstro), oligodendrocyte precursors (OPC), dentate gyrus granule neurons (Granule), and the subiculum and pyramidal neurons (CA1-sub, CA2-3-4). Using the dynamic model, we calculated the RNA velocities on cell points in a 2D UMAP representation. The streamline plot of the velocity field, along with its curl-free, divergence-free, and harmonic components, is illustrated in the last row of Figure 2.

One can see from the original velocity field that the streamlines exhibit a clear flow, originating from the radial glia, going through the intermediate stages, and finally pointing toward the five terminal stages. This cell dynamic information can also be seen in its decomposed components, especially in the harmonic component, where the streamlines clearly depict the overall cell dynamic lineage direction. Circular shapes observed in the divergence-free plot, however, do not correspond to cell cycles as the streamlines in the harmonic component related to these cell types are unidirectional.

## 3 Discussion

### 3.1 Reliability

**Figure 3:**
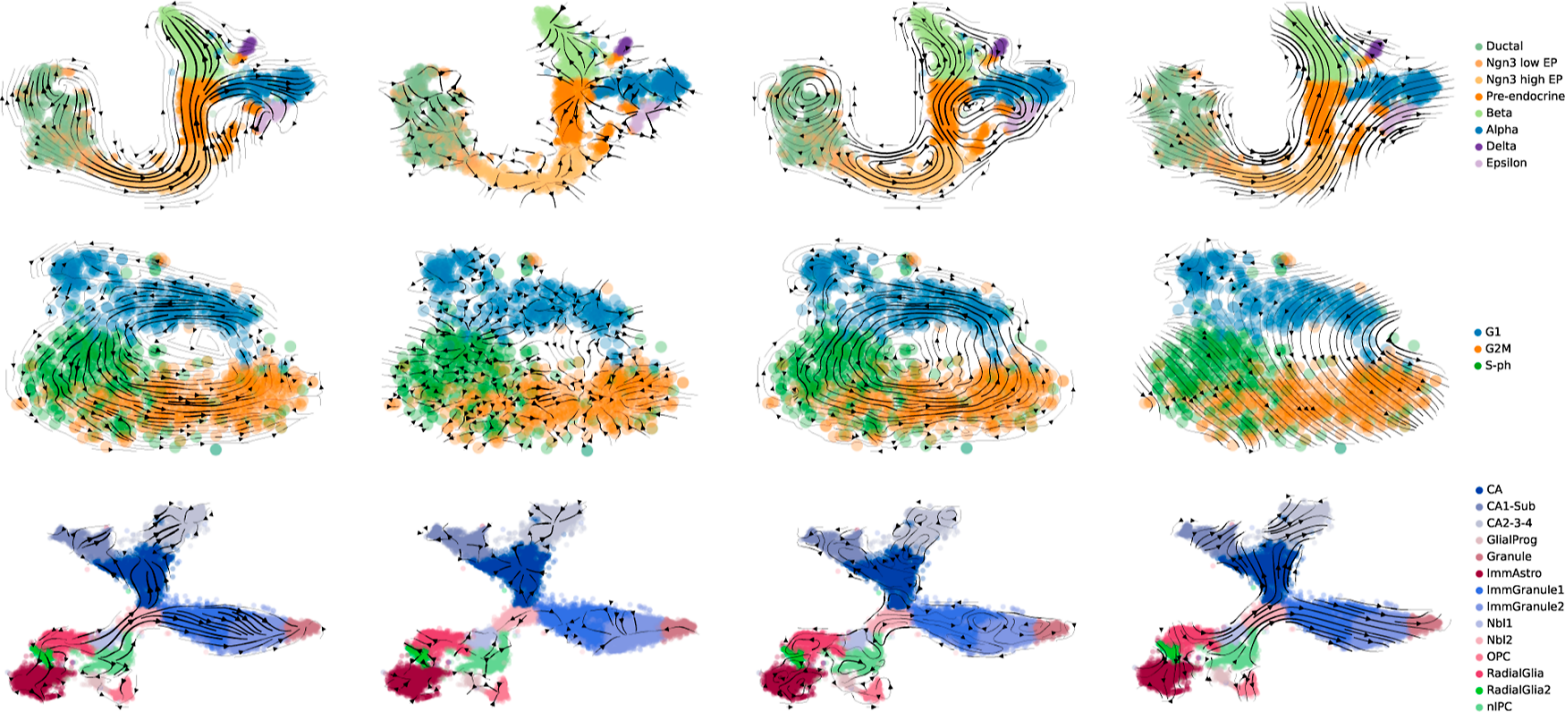
Hodge decomposition of velocity fields in 2D t-SNE representations for the pancreatic endocrinogenesis data set (top row), the cell cycle phase data set (middle row), and the developing mouse hippocampus data set (bottom row). Each row, from left to right, presents the original velocity field, its curl-free component, its divergence-free component, and its harmonic component. Here, color represents different cell types or cell phases.

The Hodge decomposition results for certain scRNA-seq data sets in their 2D UMAP representations show that the important features, such as cell cycle, bifurcation, and cell lineage differentiation, could be effectively extracted and presented on their decomposed components. To validate the reliability of the Hodge decomposition on scRNA-seq data, we considered another commonly used dimension reduction method, i.e., t-SNE, and studied the decomposed components of the projected velocity fields in 2D t-SNE representations for these scRNA-seq data sets. Corresponding streamline plots of these velocity fields on cell points and their decomposed components are presented in Figure 3. One can see that for each data set, the extracted components of the original velocity field in its t-SNE representation reveal the same cell dynamic information as we show on the decomposed components of the velocity fields projected onto the UMAP representation in Section 2. The divergence-free component reveals the cell cycle dynamics, and the harmonic component captures the overall direction of cell lineage differentiation. The presence of saddle points in the harmonic component can serve as indicators for the bifurcation process of the cells. The consistency of these results in different representations demonstrates the reliability of the Hodge decomposition of the RNA velocity fields of the scRNA-seq data.

### 3.2 Robustness

The two most important parameters in UMAP are n_neighbors and min_dist. These two parameters control the balance between the local and global structures in the projected UMAP embedding. Lower values of n_neighbors, i.e., a lower number of approximated nearest neighbors used for manifold approximation, preserve more local data structures, while larger values lead to more global views of the manifold. The typical range for n_neighbors is from 2 to 100. On the other hand, min_dist gives the minimum distance between points in the low-dimensional UMAP representation. Lower values result in a more clustered embedding, while larger values make the UMAP points more spread out. The range for min_dist is from 0 to 1.

**Figure 4:**
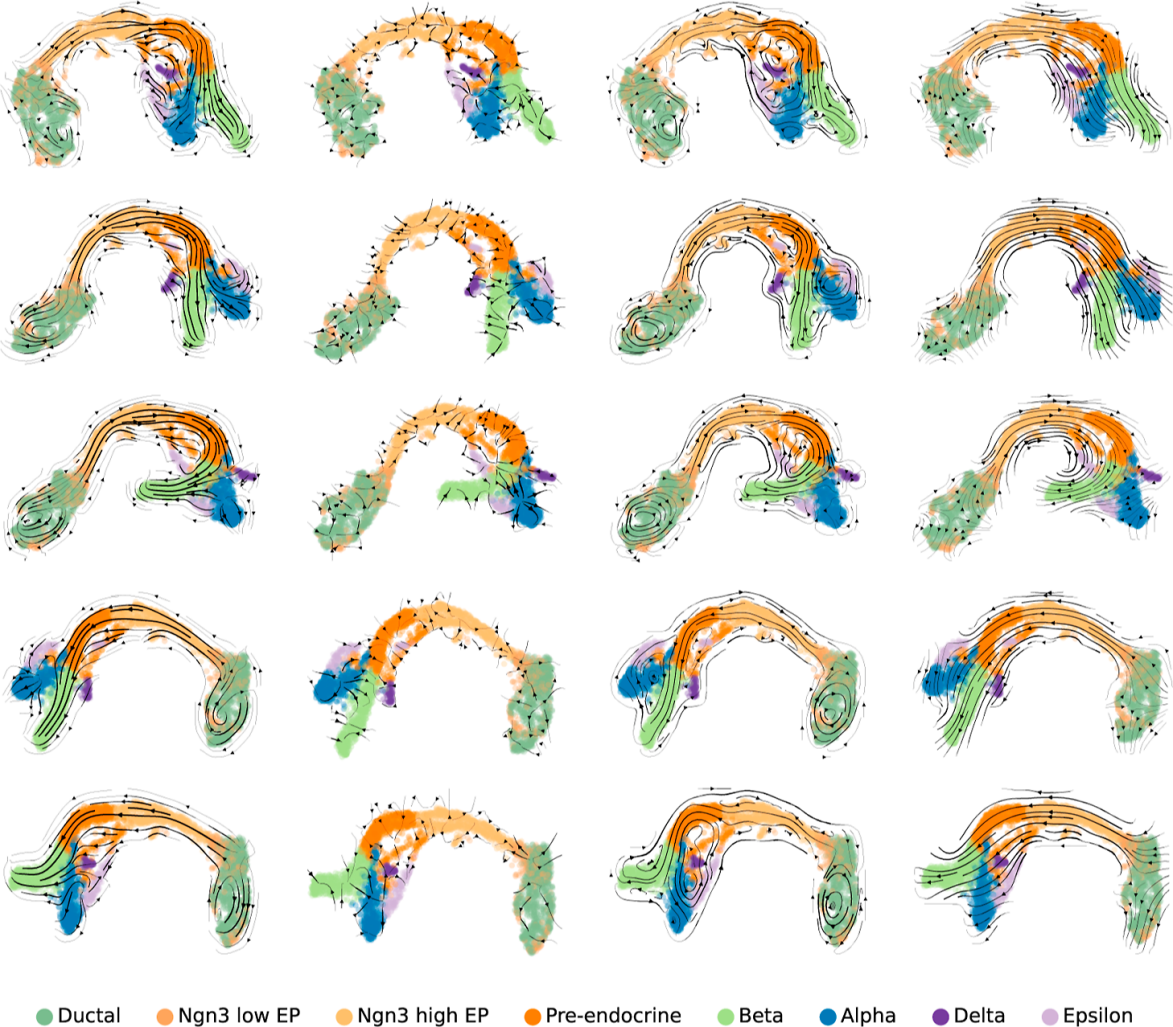
Hodge decompositions of velocity fields for the pancreatic endocrinogenesis data set with different n_neighbors in a 2D UMAP representation. Each row, from left to right, presents the original velocity field (first column), its curl-free component (second column), its divergence-free component (third column), and its harmonic component (fourth column). Rows from top to bottom correspond to n_neighbors 10, 30, 50, 70, and 90. Here, color represents different cell types.

**Figure 5:**
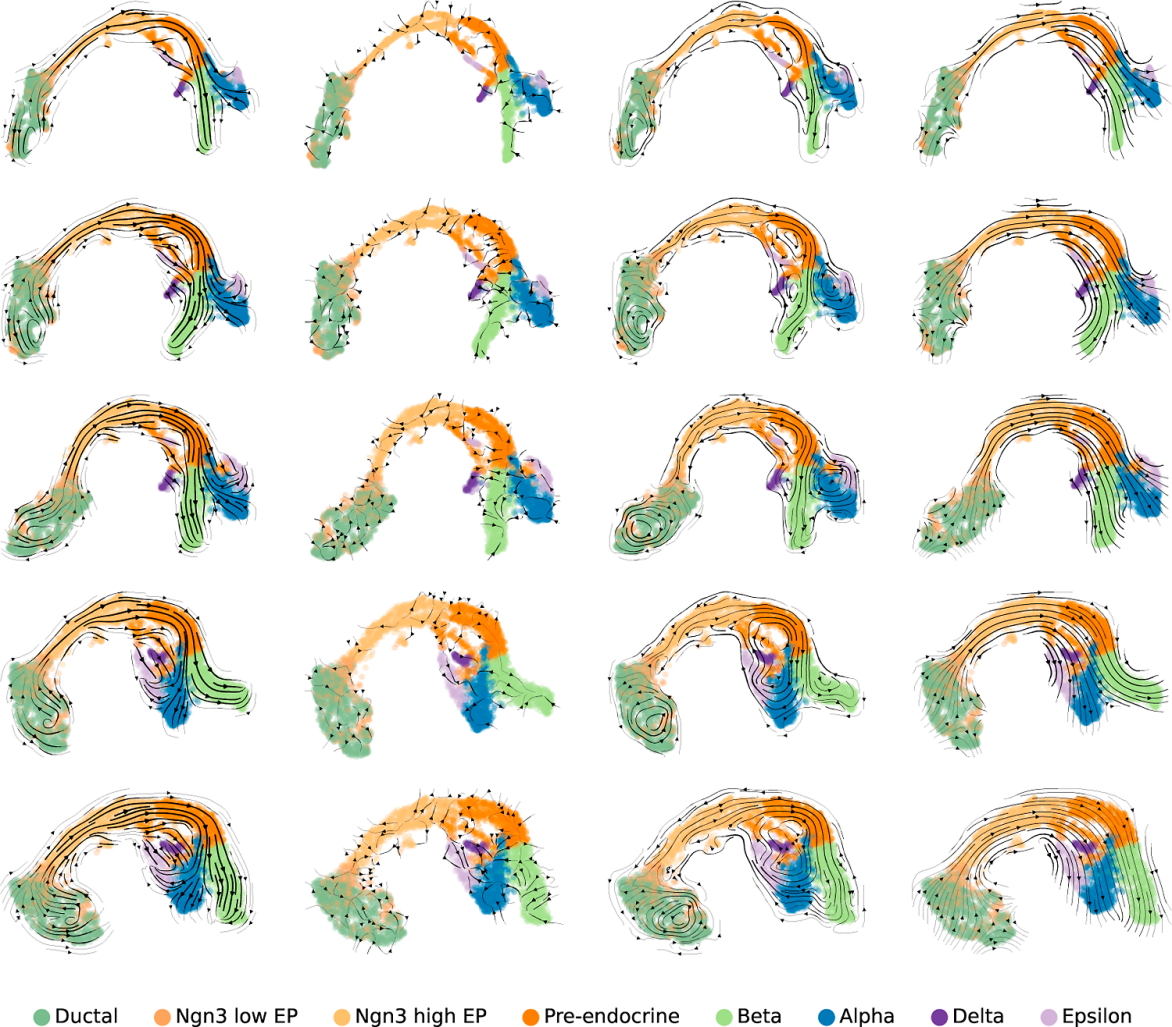
Hodge decompositions of velocity fields for the pancreatic endocrinogenesis data set with varied min_dists in a 2D UMAP representation. Each row, from left to right, presents the original velocity field (first column), its curl-free component (second column), its divergence-free component (third column), and its harmonic component (fourth column). Rows from top to bottom correspond to min_dist values 0.1, 0.3, 0.5, 0.7, and 0.9. Here, color represents different cell types.

To demonstrate the robustness of the features extracted from the Hodge decomposition of the RNA velocity fields, we employed the pancreas data set^12^ in a 2D UMAP representation, which consists of both the cycle dynamic and the cell lineage differentiation process. By adjusting one of these two parameters while keeping the other at the default value provided by scVelo, we projected the cell points and the RNA velocity fields in 2D UMAP representations obtained using the dynamic model and then applied the Hodge decomposition to the corresponding vector fields on bounded manifolds covering all cell points. In Figure 4, rows from top to bottom illustrate the decomposed results of different velocity fields on UMAP representations with n_neighbors set to 10, 30, 50, 70, and 90, while min_dist was fixed to 0.5. In Figure 5, rows, from top to bottom, present the Hodge decomposition results of different velocity fields with min_dist set to 0.1, 0.3, 0.5, 0.7, and 0.9, while n_neighbor is fixed at 30. Notably, all these different values of n_neighbors and min_dist yield consistent feature extraction results for the decomposed components.

### 3.3 3D Modeling and Future Directions

**Figure 6:**
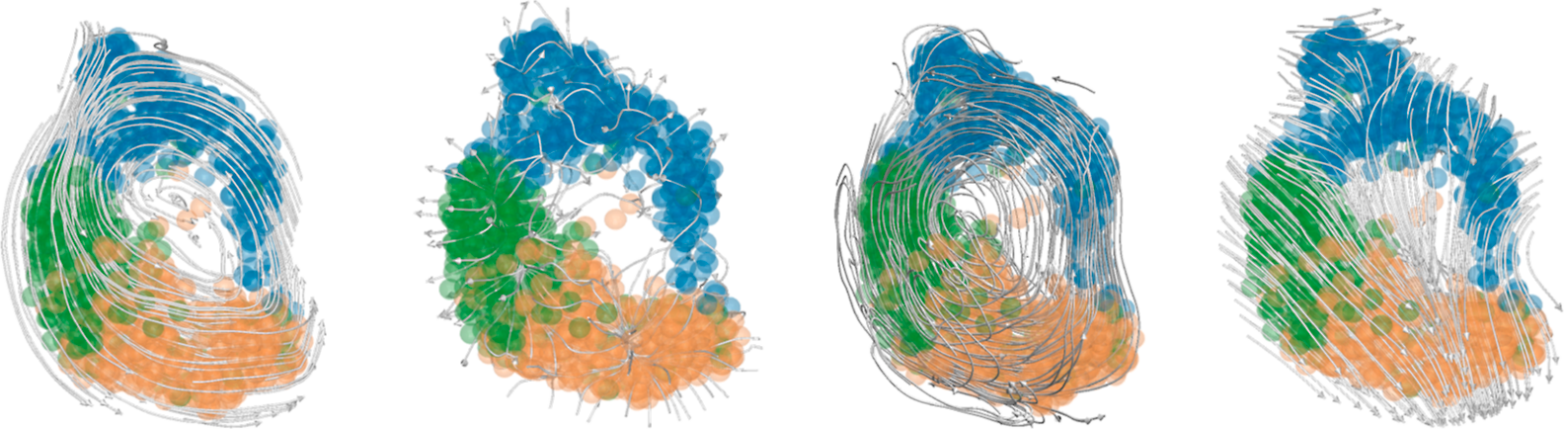
Hodge decomposition of velocity field for the cell cycle data set in a 3D UMAP representation. We have, from left to right, the original velocity field, its curl-free component, its divergence-free component, and its harmonic component. Color represents different cell phases: G1 (blue), S-Ph (green), and G2M (yellow).

In the previous sections, we focused on the Hodge decomposition of velocity fields in 2D UMAP or t-SNE representations for scRNA-seq data sets, as the 2D decomposition results for each data set in the paper already capture the key features related to the known biological processes, and the 3D results are similar to that. However, one limitation of the 2D UMAP visualizations is the potential overlap of clusters that makes points in different clusters indistinguishable. Using the 3D representation may resolve this issue while still encoding the same information as the original data. In Figure 6, we present one example of the Hodge decomposition results of the velocity field for the cell cycle phase data set^40^ in a 3D UMAP representation. One can see that in the 3D case each decomposed component of the original velocity field characterizes the same dynamic information as one has seen in the 2D case.

Furthermore, as indicated in Section 4.2.1, a vector field on a bounded domain in the Euclidean space can be further orthogonally decomposed into five components. Utilizing the 5-component Hodge decomposition might unveil more dynamic information on the original RNA velocity field. We leave all of these technical issues for further work.

## 4 Methods

Let *M* be an *m*-dimensional smooth orientable compact manifold with boundary and let Ω^*k*^(*M*) be the space of all differential *k*-forms on *M*, i.e., the set of smooth sections ∧^*k*^(T*M) of the *k*-th exterior power of the cotangent bundle T*M. The space Ω^*k*^(*M*) is a Hilbert space, where each element is an antisymmetric covariant tensor field of degree *k* on *M*. When restricted to each point, *x* ∈ *M*, a *k*-form is an antisymmetric multilinear transformation that maps *k*-vectors to scalars. A differential *k*-form can be integrated over a *k*-dimensional domain. Let us consider ω ∈ Ω^*k*^(*M*). One of the most elegant theorems concerning the integration of differential forms is Stokes’ theorem, which, as a generalization to the fundamental theorem of calculus, states that the integral of a differential *k*-form over the boundary  of some *k* + 1 submanifold *S* ⊂ *M* is equal to the integral of its exterior derivative over *S*, i.e.
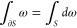
4.1where the operator *d* represents the differential (i.e., exterior derivative) that maps *k*-forms to (*k* + 1)-forms on *M*. The exterior derivative *d* is a generalization of the ordinary differential for smooth functions, and it is the unique -linear map satisfying the Leibniz rule with respect to the wedge product ∧, a generalization of the cross product for vectors, and the property dd = 0. Using the exterior derivative, the notions of closed and exact differential forms can be defined. We call a differential form ω ∈ Ω^*k*^(*M*) closed if *d*ω = 0 and exact if there is a (*k* – 1)-form ζ ∈ Ω^*k*–1^(*M*) such that ω = *d*ζ. Because dd = 0, every exact form is closed.

Given a Riemannian metric *g* on *M*, the Hodge star can be defined as the unique linear operator ★: Ω^*k*^(*M*) → Ω^*n*–*k*^(*M*) sending a *k*-form to its dual *n* – *k* form on *M* satisfying
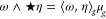
4.2for all differential *k*-forms ω and η on *M*, where ⟨·,·⟩_*g*_, for each point *p* ∈ *M*, is the inner product induced by *g* on  and μ_*g*_ is the volume form on *M* induced by the metric *g*. By taking the integral of the formula 4.2, we obtain the Hodge *L*^2^-inner product on the space of differential *k*-forms Ω^*k*^(*M*)
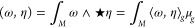
4.3which makes Ω^*k*^(*M*) a Hilbert space.

The codifferential operator is a linear map δ: Ω^*k*^(*M*) → Ω^*k*–1^(*M*) defined by
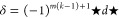
4.4which also has property that δδ = 0. A differential form ω ∈ Ω^*k*^(*M*) is called coclosed if δω = 0 and called coexact if there is a (*k* + 1)-form η ∈ Ω^*k*+1^(*M*) such that ω = δη.

### 4.1 Hodge Decomposition for Closed Manifolds

In this section, we assume *M* to be a closed manifold, i.e., *M* is a compact manifold without boundary. Let Δ = *d*δ + δ*d* = (*d* + δ)^2^: Ω^*k*^(*M*) → Ω^*k*^(*M*) be the Hodge Laplacian defined for differential forms, which is a generalization of the Laplace-Beltrami operator for functions on Riemannian manifolds. A *k*-form ω is called harmonic if it is in the kernel of the Hodge Laplacian, i.e., Δω = 0. We denote by  the space of all harmonic *k*-forms on *M*. The usual Hodge decomposition theorem^30^ tells us that there is a decomposition of the space of differential *k*-forms
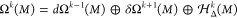
4.5

These three subspaces are orthogonal, with respect to the inner product (eq 4.3). Thus, any *k*-form on a closed manifold *M* can be written as a unique sum of an exact form, a coexact form, and a harmonic form
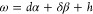
4.6

The orthogonality of these three terms follows directly from the adjointness of the differential *d* and the codifferential δ
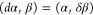
4.7and the identification
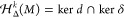
4.8

The adjointness (eq 4.7) comes from integration by parts and the boundarylessness of the manifold, and it leads directly to the following formula
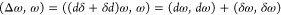
4.9

It is easy to see that a *k*-form is harmonic if and only if it is both closed and coclosed, which immediately produces the identification (eq 4.8).

*H*_*dR*_^*k*^(*M*) denotes the *k*-th de Rham cohomology group, which is defined by the set of closed forms in Ω^*k*^(*M*) modulo the exact forms, i.e., *H*_*dR*_^*k*^(*M*) = ker *d*^*k*^/Im *d*^*k*–1^. For closed manifolds, the Hodge theorem identifies *H*_*dR*_^*k*^(*M*) with the space of harmonic *k*-forms , which states that each equivalence class in *H*_*dR*_^*k*^(*M*) contains exactly one harmonic form. In addition, it follows from the de Rham theorem and Poincaré duality that the de Rham cohomology group *H*_*dR*_^*k*^(*M*) is isomorphic to the (*m* – *k*)-singular homology group, the dimension of which is given by the (*m* – *k*)-th Betti number. As a consequence, the harmonic part depends only on the topology of *M*.

### 4.2 Hodge Decomposition for Manifolds with Boundary

The situation becomes complicated for manifolds with a boundary, as some additional requirements on the boundary are necessary. While the total vector field can be given with arbitrary boundary values, we restrict component subspaces of differential forms Ω^*k*^(*M*) to those satisfying certain boundary conditions. These boundary conditions ensure that we have the adjointness of differential *d* and codifferential δ, thereby guaranteeing an orthogonal decomposition of the space of *k*-forms. Moreover, the correspondence to the topology can only be established with proper choices on boundary conditions.

Two common choices of boundary conditions consistent with physical boundary conditions and ensuring the adjointness of differential *d* and codifferential δ are the normal (Dirichlet) boundary condition and tangential (Neumann) boundary condition. We call a form ω ∈ Ω^*k*^(*M*) normal (Dirichlet forms) if it gives zero when applying to the tangent vectors of the boundary, and tangential (Neumann forms) if its dual ★ ω is zero when applying to the tangent vectors of the boundary. Ω_*n*_^*k*^(*M*) denotes the set of normal *k*-forms and Ω_*t*_^*k*^(*M*) denotes the set of tangential *k*-forms in Ω^*k*^(*M*). We have
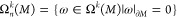
4.10
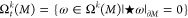
4.11

It can be seen from the definitions of the spaces of normal forms and tangential forms that Hodge star ★ provides an isomorphism between the two spaces Ω_*n*_^*k*^(*M*) and Ω_*t*_^*m*–*k*^(*M*). Furthermore, by restricting the differential *d* to the boundary , we can see that *d* preserves the normal boundary condition and the codifferential operator δ preserves the tangential boundary condition.

The space of differential *k*-forms Ω^*k*^(*M*) can then be orthogonally decomposed into three subspaces^42^ (Hodge–Morrey decomposition) as follows
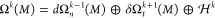
4.12where , called the space of harmonic fields,^43^ is the space of differential *k*-forms that are both closed and coclosed. The space of harmonic fields  is infinite-dimensional in general. It is easy to see that every harmonic field is a harmonic form, i.e., Δω = 0, but the converse is no longer true. The orthogonality comes from the following formula
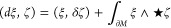
4.13for any (*k* – 1)-form ξ and *k*-form ζ with arbitrary *k*. When either ξ is normal or ζ is tangential, the integral term over the boundary  vanishes, leading to the adjointness of differential *d* and codifferential δ. The orthogonality of the three terms with respect to the inner product (eq 4.3) then follows immediately.

It is worth noting that in the case of closed manifolds, the space of normal differential forms Ω_*n*_^*k*^(*M*) and the space of tangential differentials Ω_*t*_^*k*^(*M*) both reduce to the space of differential forms Ω^*k*^(*M*). Thus, the decomposition in formula 4.12 simplifies to exactly the one (eq 4.5) for closed manifolds.

#### 4.2.1 Remark 4.1

Let  denote the space of normal harmonic differential *k*-forms and  denote the space of tangential differential *k*-forms. The space  can actually be further decomposed into three terms^44^
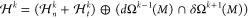
4.14

Note that in general, the space  and  are not orthogonal with respect to the inner product (eq 4.3). However, they are orthogonal for domains in .^31^ In addition, the normal harmonic space  is isomorphic to the relative de Rham cohomology *H*_*dR*_^*k*^(*M*,∂*M*), and the tangential space  is isomorphic to the absolute de Rham cohomology *H*_*dR*_^*k*^(*M*),^44^ and thus they are both finite-dimensional. The dimensions of these spaces are given by the Betti numbers  and , which are fully determined by the topology of the manifold .

Given a differential *k*-form ω ∈ Ω^*k*^(*M*), by the Hodge decomposition (eq 4.12) we have the following unique representation
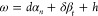
4.15where α_*n*_∈Ω_*n*_^*k*–1^(*M*), β_*t*_∈Ω_*t*_^*k*+1^, and . To compute the three terms in the decomposition, we need to solve for the potentials α and β and then apply differential and codifferential operators to the potentials α and β to obtain the first two terms. The harmonic term is then calculated by subtracting the first two terms from the differential *k*-form ω. However, the potentials are usually not uniquely determined, as all α + *d*η and β + δγ for η ∈ Ω^*k*–2^(*M*) and γ ∈ Ω^*k*+2^(*M*) serve as potentials for the same input. To address this, we apply a variety of gauge conditions ensuring the uniqueness of potentials by restricting solutions to the space of coclosed normal (*k* – 1)-forms Ω_*n*_^*k*–1^(*M*) ∩ ker *δ* for α, and the space of closed tangential (*k* + 1)-forms Ω_*t*_^*k*+1^(*M*) ∩ ker *d* for β. By applying independently the codifferential δ and the differential *d* to (eq 4.15), we have
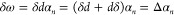
4.16
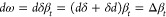
4.17

The potentials can then be calculated by these two eq 4.16 by enforcing extra boundary conditions δα_*n*_|_∂*M*_ = 0, and ★*d*β_*t*_|_∂*M*_ = 0, and resolving the rank deficiencies β_*m*–*k*+1_ and β_*k*+1_.^21^

In this work, we choose DEC^32^ as our tool to represent all differential operators and differential forms for computations. The dual mesh structure in DEC helps in constructing discrete differential operators and differential forms that preserve Stokes’ theorem, approximate their continuous analogues, and allow for robust and accurate numerical algorithms. For efficient discretization of the manifold *M*, we simply represent the manifold *M* as a bound region with vertices all fixed on a regular Cartesian grid. Its dual is then given by the translated grid with vertices at the centers of cells of the original grid.

By utilizing DEC, eq 4.16 can be solved as a sparse linear system with discrete Laplacians. Applying the discrete differential and codifferential operators to the two obtained discrete potentials α_*n*_ and β_*t*_ produces the exact and the coexact components in eq 4.15. The discrete harmonic component can subsequently be calculated by subtracting these two components from the original differential form ω.

### 4.3 Helmholtz–Hodge Decomposition

In the case that *M* is a compact domain in  or 3, equipped with the standard Euclidean metric, the differential forms reduce to scalar or vector fields depending on their degrees. For instance, in , following from the isomorphisms of the sharp and flat operators, a 0-form or 3-form can be identified with a scalar field, and a 1-form or 2-form can be regarded as a vector field. In addition, we have the correspondences between the differential operators *d* and codifferential operators δ and gradient, curl, and divergence, denoted by ∇, ∇×, and ∇·, on 0-forms, 1-forms, and 2-forms, respectively. Following from dd = 0, we have ∇ × ∇ = 0 and ∇·∇× = 0. A vector field **v** is called curl-free if its curl gives 0, i.e., ∇ × **v** = 0, and divergence-free if its divergence gives 0, i.e., ∇·**v** = 0. The HHD for vector fields in  (seen as 1-forms) is given as follows
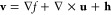
4.18where **v** is a vector field defined on *M*, *f* is a scalar potential on *M* that vanishes on the boundary ∂*M*, **u** is a vector field parallel to the normal direction of the boundary, and **h** is the harmonic vector field satisfying ∇ × **h** = 0 and ∇·**h** = 0. The first component ∇*f* and the second component ∇ × **u** are called the curl-free part and the divergence-free part of **v**.

In the two-dimensional case, the curl operator is just a scalar quantity in the upward normal direction of the plane. The second term ∇ × *u* in (eq 4.18) can be simplified to *J*∇*u*, where *J* rotates a 2D vector counterclockwise by π/2. We then have the HHD for vector fields in 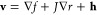
4.19where both *f* and *r* are scalar potentials that vanish on the boundary ∂*M*.

The HHDs are direct counterparts of the Hodge decomposition (eq 4.12) for vector fields defined on a bounded domain *M* in  or . A complete correspondence between differential forms and vector fields under the normal and tangential boundary conditions in dimension 3 can be found in.^21^ The three terms in (eq 4.18) or (eq 4.19) correspond exactly to the three terms in the Hodge decomposition (eq 4.12) in the same order. Therefore, by representing a vector field as a 1- or 2-form on *M*, the Hodge decomposition (eq 4.15) can be straightforwardly implemented through DEC on Cartesian grids^34^ to extract the curl-free component, the divergence-free component and the harmonic component of the original vector field.

### 4.4 Data Preprocessing and Manifold Generation

The analysis of RNA velocities requires two gene-by-cell count matrices representing the unspliced mRNA abundances and the spliced (mRNA) abundances. Several pipelines such as velocyto,^9^ kallisto-bustools,^45^ STARsolo,^46^ alevin,^47^ and alevin-fry^48^ have been introduced to generate these two count matrices from the original scRNA-seq data. However, the scRNA-seq data are noisy and highly variable. Preprocessing the data is necessary and may involve steps such as selecting high-variable genes or removing low-quality cells, normalizing the data by their gene size to enable accurate expression comparisons between cells, and log-transforming to stabilize the variance. One can then calculate its low dimensional representation of cell points along with the corresponding velocities using tools like velocyto, scVelo, etc. depending on the selected RNA velocity models.

The decomposition of vector fields on a Cartesian grid requires a vector field defined on all grid points within a bounded region in a two-dimensional (2D) or three-dimensional (3D) Cartesian grid. Since the decomposition of a vector field is independent of the choice of the grid. We can just choose a tight bounding box that contains all of the cell points in their low-dimensional representation and then create a regular Cartesian grid by subdividing each edge of the box into subsegments of equal length, referred to as grid edges. To calculate the vector fields defined on all vertices (grid points), we employ an unsupervised nearest neighbors learning method provided by Scikit-learn to identify its nearest 50 cell points for each grid point and then compute the velocity at this grid point by taking the weighted average of the velocities at these nearest cell points, with weights determined by a Gaussian kernel.

We are not interested in the grid points that are far away from the cell points, as these points and the velocities defined on them do not hold any significance for our scRNaseq analysis. To restrict ourselves to a domain that covers the cell points, we utilize the flexibility rigidity index (FRI) density function^49,50^ to create a continuous distribution defined in the grid, and then choose carefully an isovalue such that all cell points are contained in the domain *M* bounded by the corresponding density function isocurve in 2D or isosurface in 3D. The isocurve or the isosurface then serves as the boundary of the domain *M*. Let {**x**_*i*_, *i* = 1, ..., *s*} be the coordinates of a set of cell points. The formula of the FRI density function is given as follows^49,50^
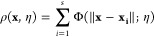
4.20where the correlation function Φ satisfies the following admissibility conditions
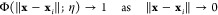
4.21
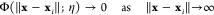
4.22

The FRI density has been shown computationally stable in translating the discrete representation of point clouds to a continuous embedding,^49,50^ for example, in generating protein boundary surfaces.^51^ There are many choices of correlation functions Φ that could be used here in the FRI density function for generating the boundary surface in the grid, such as the generalized exponential functions, the generalized Lorentz functions, etc. A detailed description of the choices on these correlation functions can be found in.^49^ In this work, we use the Gaussian function on  or  as the correlation function Φ with formula
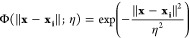
4.23

The scale η can be selected in accordance with the data. In this paper, we simply set η = 1. With a proper isovalue *c*, the manifold *M* containing all cell points in the grid is of the following representation
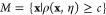
4.24with its boundary being the isocurve or isosurface ∂*M* = {**x**|ρ(**x**,η) = *c*}.

## 5 Conclusions

The RNA velocity is able to effectively unveil the dynamic information on cells in a biological process through the velocity fields, where the corresponding features can be easily visualized by examining circular shapes and the flow directions in the velocity plot embedded in a low-dimensional representation obtained using UMAP or t-SNE. By the Hodge decomposition, one can extract these dynamic features in the resulting decomposed components of the original velocity field, for example, the circular shapes in its divergent-free component that are associated with the cell cycle process and the overall flow direction in the harmonic component that provides the cell lineage information. In addition, the bifurcation process is indicated by saddle points in the harmonic component, given as a more apparent visualization form than that on the original velocity field, showing the unstable state of the cells. The curl-free, divergence-free, and harmonic components of the RNA velocity fields have the potential to be further studied and analyzed to unveil additional information on the cell dynamics. Just as in,^13^ the study of geometric features of each component, such as magnitude, acceleration, and curvature, can enhance the understanding of cell dynamics for single-cell data sets.

The Hodge decomposition for manifolds with boundary provides a powerful mathematical tool for extracting the curl-free, divergence-free, and harmonic components of a vector field by imposing proper boundary conditions. The numerical algorithms, facilitated by DEC, can be easily implemented and efficiently computed by relying on just matrix algebra. The reliability and robustness of the present analysis have been analyzed. We expect that the present Hodge decomposition of scRNA-seq velocity fields offers a new analytical tool to gain insights into cell dynamics in biological processes.

## Data Availability

The data sets used in this paper are publicly released and published. The raw data set of pancreatic endocrinogenesis is available from the Gene Expression Omnibus (GEO) under accession GSE132188. The raw data set of hippocampal dentate gyrus neurogenesis is available at GEO accession GSE95753. The raw data set of the cell cycle phase is available at GEO accession GEOGSE146773. The Matlab code for Hodge decomposition can be found in the following GitHub repository: https://github.com/WeilabMSU/HHD.
